# Dynamics of mitochondrial heteroplasmy in three families investigated via a repeatable re-sequencing study

**DOI:** 10.1186/gb-2011-12-6-r59

**Published:** 2011-06-23

**Authors:** Hiroki Goto, Benjamin Dickins, Enis Afgan, Ian M Paul, James Taylor, Kateryna D Makova, Anton Nekrutenko

**Affiliations:** 1 The Huck Institutes of Life Sciences and Department of Biology, Penn State University, 305 Wartik Lab, University Park, PA 16802, USA; 2 The Huck Institutes for the Life Sciences and Department of Biochemistry and Molecular Biology, Penn State University, Wartik 505, University Park, PA 16802, USA; 3 Departments of Biology and Mathematics & Computer Science, Emory University, 1510 Clifton Road NE, Room 2006, Atlanta, GA 30322, USA; 4 Department of Pediatrics, Penn State College of Medicine, 500 University Drive, Hershey, PA 17033, USA

## Abstract

**Background:**

Originally believed to be a rare phenomenon, heteroplasmy - the presence of more than one mitochondrial DNA (mtDNA) variant within a cell, tissue, or individual - is emerging as an important component of eukaryotic genetic diversity. Heteroplasmies can be used as genetic markers in applications ranging from forensics to cancer diagnostics. Yet the frequency of heteroplasmic alleles may vary from generation to generation due to the bottleneck occurring during oogenesis. Therefore, to understand the alterations in allele frequencies at heteroplasmic sites, it is of critical importance to investigate the dynamics of maternal mtDNA transmission.

**Results:**

Here we sequenced, at high coverage, mtDNA from blood and buccal tissues of nine individuals from three families with a total of six maternal transmission events. Using simulations and re-sequencing of clonal DNA, we devised a set of criteria for detecting polymorphic sites in heterogeneous genetic samples that is resistant to the noise originating from massively parallel sequencing technologies. Application of these criteria to nine human mtDNA samples revealed four heteroplasmic sites.

**Conclusions:**

Our results suggest that the incidence of heteroplasmy may be lower than estimated in some other recent re-sequencing studies, and that mtDNA allelic frequencies differ significantly both between tissues of the same individual and between a mother and her offspring. We designed our study in such a way that the complete analysis described here can be repeated by anyone either at our site or directly on the Amazon Cloud. Our computational pipeline can be easily modified to accommodate other applications, such as viral re-sequencing.

## Background

The mitochondrial genome is maternally inherited and harbors 37 genes in a circular molecule of approximately 16.6 kb that is present in hundreds to thousands of copies per cell [1] and has accumulated mutations at a rate at least an order of magnitude higher than its nuclear counterpart [2,3]. Frequently, more than one mtDNA variant is present in the same individual, a phenomenon called 'heteroplasmy' [4]. The mitochondrial genome is implicated in hundreds of diseases (over 200 catalogued at [5] as of mid-2010) with the majority of them caused by point mutations [6]. Multiple mtDNA mutations might also predispose one to common metabolic and neurological diseases of advanced age, such as diabetes as well as Parkinson's and Alzheimer's diseases [7]. Additionally, mtDNA mutations appear to have a role in cancer etiology [8]. Many disease-causing mtDNA variants are heteroplasmic and their clinical manifestation depends on the relative proportion of mutant versus normal mitochondrial genomes [7,9,10]. No effective treatment for genetic diseases caused by mtDNA mutations currently exists, placing great emphasis on reducing the occurrence and preventing the transmission of these mutations in human populations [11]. There is therefore a pressing need to understand the biological mechanisms for the origin and transmission of heteroplasmic mtDNA mutations. In addition, mtDNA has been widely used as a marker in molecular evolution, population genetics and forensics. So, unraveling the dynamics of heteroplasmic mtDNA mutations will have important impacts for these fields. It is known that mtDNA genomes undergo a bottleneck (decrease in numbers) during oogenesis; however, the exact size of this bottleneck in humans, likely to be different from that in mice, has been disputed and is not easily amenable to experimental estimation [12]. Knowledge of the size of the bottleneck is critical for modeling mtDNA evolution, assessing its applicability as a genetic marker, and for genetic counseling of patients carrying mtDNA mutations [13]. The size of the mtDNA bottleneck can be estimated more accurately when low frequency heteroplasmic mutations are taken into account [14].

In this study we pursued two goals. First, we wanted to develop a robust workflow for detection of heteroplasmies from next-generation sequencing (NGS) data and use it to trace maternal transmission events. This is because, despite the apparent importance of the mutational dynamics of mtDNA, our understanding of this process is hampered by lack of resolution, as most published studies have used capillary sequencing that can accurately detect only heteroplasmies with frequencies >20% [15]. Therefore, some mutations detected in such a manner were not real mutations, but shifts in heteroplasmy frequency between generations (for example, from 15% in a mother to 85% in a child), and other cases of real *de novo *mutations might have gone undetected (for example, from 0% in a mother to 10% in a child). The development and continuing evolution of sequencing technologies offer a unique opportunity to overcome these hurdles. Two recent studies have used Illumina sequencing technology to study mtDNA heteroplasmy in normal and cancerous tissues [16,17]. The first study [16] concluded that heteroplasmy affects the entire mitochondrial genome and is common in normal individuals. Additionally, these authors analyzed cell lines derived from individuals of two families and suggested that most heteroplasmic mutations arise during early embryogenesis. However, because only lymphoid cell lines were analyzed, some of these mutations might have either been germline (and not somatic) or arisen during expansion of lymphoid cells in culture. In the second study [17], the authors put a significant effort into the investigation of limitations associated with calling heteroplasmic variants from re-sequencing data generated by Illumina platform. They sounded a cautionary note after finding a relatively small number of variable sites (37 sites in 131 unrelated individuals) and pointing out that some variants reported by [16] might arise from artifacts of Illumina sequencing. The discrepancy between the two studies underscores the fact that, despite the much higher resolution provided by Illumina platform (and other NGS technologies), the detection of heteroplasmic variants requires robust approaches such as the one we sought to develop here.

The second goal of this study was to design our analyses in such a way that they can be easily repeated by others. Reproducibility is particularly important if heteroplasmies are to be used as markers in applications such as cancer diagnostics, as suggested by [16]. In fact, the concern over reproducibility is common to almost all studies utilizing the NGS technology. As mentioned above, the advantage of using NGS for re-sequencing lies in multiple sampling of individual genomic positions by numerous independent reads, allowing for reliable detection of very rare variants. Although conceptually analysis of re-sequencing data is straightforward - collect the data and map the reads - there are no established practices for performing such analyses that can be adopted easily by computationally averse investigators comprising the majority of biomedical researchers. This is largely due to the novelty of NGS technology as well as its continuing rapid evolution and proliferation. Because new tools for the analysis of NGS data appear on a monthly basis, it is more important than ever to preserve primary datasets, for they may be re-analyzed as new algorithms are implemented. To alleviate this difficulty, we designed our study in such a way that anyone can reproduce our analyses in their entirety, modify them, or tailor them to his/her specific needs as described at [18].

## Results and discussion

### Families, tissues, and sequencing

**Figure 1:**
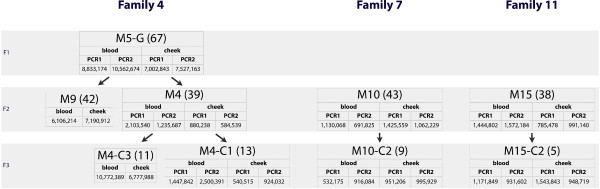
**Individuals and samples used in the study**. Numbers in parenthesis are the age of each individual; the number at the bottom of each table is count of sequencing reads.

As a pilot dataset for our study, we chose nine individuals from three families representing six mother-to-child transmission events (Figure 1). For each individual, the DNA was collected from a cheek swab specimen and from blood by our clinical collaborators at Penn State College of Medicine, and mitochondrial genomes were amplified by PCR using two primer pairs (see Materials and Methods). To control for possible PCR-induced errors, each amplification was performed twice (with the exception of individuals M9 and M4-C3, for which a single PCR was performed per tissue). In total we generated (7 individuals × 2 tissues × 2 PCRs) + (2 individuals × 2 tissues × 1 PCR) = 32 single-end 76-bp (100-bp reads were generated for blood of M4, M9, and M4-C3) Illumina datasets (Figure 1). After generating consensus sequences for each sample based on the hg19 reference (AF347015), we adjusted the indexing to the Cambridge Reference Sequence (NC_012920), collated SNPs (indels were not accounted for) and determined the haplogroups using the HaploGrep algorithm incorporating Phylotree version 11 [17]. We determined that members of families 4, 7, and 11 belong to haplogroups H1, U3a1 and K2a, respectively.

### A robust set of criteria for detection of mitochondrial variation

Even with the vast coverage that can be achieved with modern sequencing technologies, detection of mitochondrial heteroplasmic sites is a challenge, for it is often difficult to distinguish between true allelic sites and sequencing errors. To date, the methodologies for the detection of heteroplasmic variants from NGS data can be distilled from a simple counting of variants after aligning reads to a reference and application of various thresholds to these counts in an attempt to weed out the noise. In the most straightforward case described by He *et al*. [16], the authors aligned the reads against the human genome using a standard Illumina pipeline and derived a frequency threshold (1.6%) by comparing sequencing reads from three PCR replicates. This threshold was uniformly applied to all samples and any sites with allele frequencies below 1.6% were discarded. In a more recent study, Li *et al*. [17] devised a set of criteria for reliable detection of heteroplasmy by conducting simulations, sequencing a clonal specimen (bacteriophage ϕX174) and detecting heteroplasmic sites in artificially mixed samples. In addition to deriving a sequencing coverage-dependent frequency threshold (10%, as their coverage was generally low), these authors used base quality values (phred metric [19] cutoffs of 20 and 23) and required all heteroplasmies to be validated by at least two reads on each strand. Application of this strategy to mtDNA samples from 131 individuals revealed 37 heteroplasmic sites, which is significantly fewer than the number reported by He *et al*. [16], who did not use quality filtering and double-stranded validation.

In designing our study, we adopted the strategy described in [17] by conducting simulations, sequencing a clonal specimen, using base quality values, and requiring all heteroplasmies to be validated by reads on each of the two sequenced strands. Importantly, compared with [17], we aimed at lowering the detection threshold by increasing per-base coverage in our samples. To estimate the detection threshold appropriate for our study, we first selected the dataset with the smallest number of reads (M4, cheek, PCR2, 584,539 reads; Figure 1) and mapped it against the hg19 version of the human genome with BWA mapper [20] as described in Materials and Methods. After retaining only reads that map uniquely to the mitochondrial genome, we obtained a coverage distribution with a median of 1,170× (Figure S1 in Additional file 1).

#### Simulations

**Figure 2:**
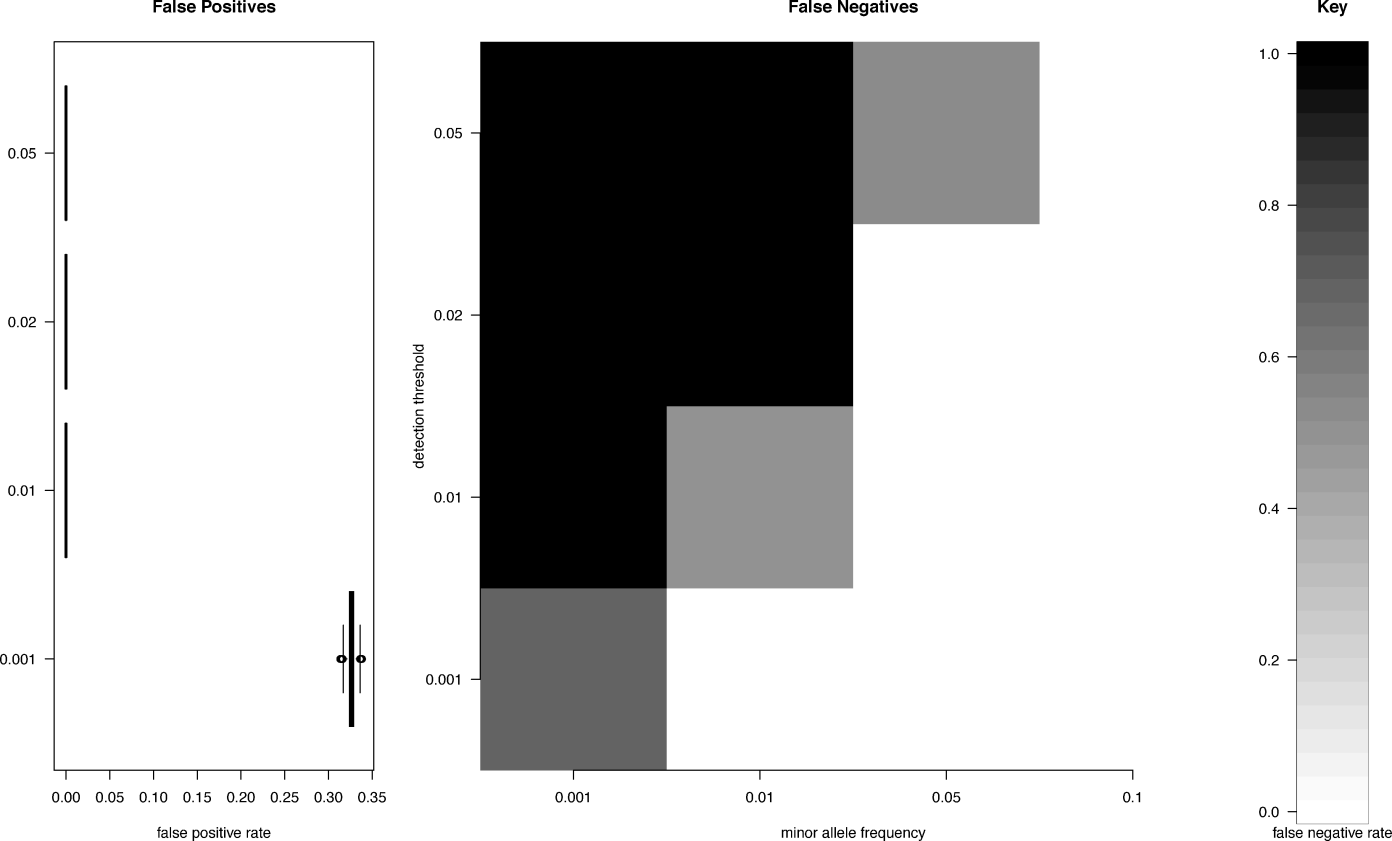
**False positive and false negative rates computed from simulation assuming 1,170× coverage**. A Python script used to generate these results can be found in Additional file 3.

Using coverage of 1,170× as a conservative starting point, we performed simulations (as described in Materials and Methods) to estimate the false positive and false negative rates given different sequencing error rate thresholds (0.001, 0.01, 0.02, and 0.05) and minor allele frequencies (heteroplasmy detection thresholds of 0.001, 0.01, 0.05, and 0.1; see Materials and Methods for the exact algorithm and the corresponding Python script). Results of these simulations are summarized in Figure 2. One can see that when the minor allele frequency and the sequencing error rate are set to 0.01 and 0.001 (the latter corresponding to a phred [19] value of 30), respectively, the resultant false negative and false positive rates are near zero. In other words, with the coverage we utilized for our sequencing, we can accurately detect heteroplasmies with the minor allele frequency above 0.01 supported by sequencing reads where the corresponding nucleotide has a quality score of at least 30 on the phred scale.

#### Sequencing a clonal specimen

Before applying these settings to our datasets, we wanted to confirm whether these hold for the real data, which we expected to be much noisier. To achieve this, we sequenced a pUC18 plasmid isolated from a single colony, which in theory should have no allelic variation ('heteroplasmies'; ϕX174 utilized by Li *et al*. [17] houses a considerable amount of variation [21] and pUC18 is a much cleaner 'non-heteroplasmic' standard, as demonstrated by the cloning and re-sequencing experiment detailed in Materials and methods). After extracting uniquely mapped reads, the coverage ranged from 19,382 × to 1,932,630 × with a median of 1,157,250×. A raw count of differences (supported by bases with quality score ≥30 on the phred scale) revealed that all positions across the plasmid contained at least two reads with deviant nucleotides (that is, different from the reference; the median number of deviant reads per position was 154), confirming considerable noise in the data. Applying the 0.01 frequency threshold derived from simulations described above eliminated all variation with the exception of site 880 (with the major allele 'G'), which contained a minor allele 'C' with the frequency of 0.025. To confirm that this is in fact a pUC18 variant (a prototype of a heteroplasmic site), we analyzed reads that mapped to forward and reverse strands separately. Such strand-specific filtering was reported by Li *et al*. [17] to eliminate the absolute majority of false positives. These authors required each variant to be confirmed by at least two reads on each strand. Here we chose to be even more conservative and required each variant to have the frequency ≥0.01 on each strand. Application of this criterion eliminated site 880, thus removing all variable sites and confirming that our criteria eradicate the noise.

#### PCR duplicates

The very high coverage in the pUC18 experiment also allowed us to evaluate the effect of PCR duplicates arising during Illumina sequencing on polymorphism detection. Such PCR duplicates usually result in a single read being repeated a large number of times. If a read subjected to PCR duplication carries a polymorphism, the frequency of this polymorphism becomes artificially inflated. The pUC18 dataset contained a large number of PCR duplicates with some reads repeated in excess of 50,000 times. However, because we require reads on both strands to validate each polymorphism, PCR duplicates did not affect our final result.

#### PCR amplification

Our experimental design allowed us to estimate the amount of error originating from PCR amplification of samples (not to be confused with PCR duplicates discussed above). Here we consider errors occurring during PCR-based enrichment of mitochondrial DNA prior to sequencing. Although Li *et al*. [17] detected no PCR-induced errors, their detection level was relatively low. To see whether amplification may potentially bias our results, we mapped all PCR replicates separately to the genome and then compared them to each other, as explained in Materials and methods (also see Additional file 2). Briefly, we were looking at all sites where one PCR replicate contained an allelic variant with a frequency ≥0.01, while the other did not contain variants at the same site. None of the samples contained such sites and therefore PCR aberrations do not create problems in our data at the 0.01 frequency threshold.

#### Final criteria for detecting heteroplasmy

The above experiments allow us to formulate a set of rules for detection of heteroplasmic sites in our samples. To call a site heteroplasmic, we require the frequency of reads supporting a particular allele to be ≥0.02 (to be conservative, we doubled the threshold from 0.01 to 0.02) on each strand and the quality of the base aligning to such a position to be ≥30 on the phred scale (corresponding to an error probability of 0.001).

#### Analysis of mixed samples: heteroplasmy recovery and score recalibration

To confirm recovery of true polymorphisms by the above set of criteria, we prepared a mix of DNA from two individuals (M4 and M10C1 from families 4 and 7, respectively) with 24 fixed single nucleotide differences (Figure S2 in Additional file 1). The mixing ratio (49:1; see Materials and methods) was set to yield a 2% apparent minor allele frequency with the identity of the minor alleles corresponding to the M10C1 sequence. In other words, the mixing was performed to make fixed differences between the two individuals appear as 'heteroplasmies' with a minor allele frequency of approximately 2%. The mixed sample was sequenced to obtain 1,713,268 140-bp single-end reads. The reads were mapped and analyzed using a procedure identical to that described below (and see [18]). All 24 'polymorphic sites' were successfully recovered with this approach (Figure S2a, b in Additional file 1). The two PCR fragments (A and B) were mixed separately, with 5 polymorphic sites in fragment A only, 17 sites in fragment B only, and 2 sites covered by both fragments. The ranges of such mixed 'heteroplasmies' are very tight, and are below our 2% threshold, arguing for the threshold validity: fragment A differences were, on average, 4.70% (median = 4.81; range = 4.02 to 5.10; data with quality score cutoff of 30); fragment B differences were, on average, 2.91% (median = 2.98; range = 2.19 to 3.55); the two sites covered by both fragments averaged 3.04% (range = 2.97 to 3.11). The resulting heteroplasmy ratios differed from 2%, but we attribute this to pipetting error.

State-of-the-art genotyping pipelines such as the one used in the 1000 Genomes Project utilize post-alignment recalibration of machine-reported base quality scores to improve the reliability of polymorphism calls. To test the effect of recalibration on our data, we applied the approach implemented in the GATK software [22] to recalibrate base qualities in reads corresponding to the mixed sample described here. Although recalibration decreased the number of bases with phred-scaled quality of 30 (Figure S3 in Additional file 1), it did not change the outcomes of our analysis, with all minor variants being reliably detected (Figure S2 in Additional file 1). Although the exact frequencies of the minor alleles changed after recalibration (Figure S2C & D in Additional file 1), the change was not significant. Indeed, in an ANOVA with ampliconic segment (A, B or overlapping, as mtDNA was amplified in two segments A and B with a small overlap), recalibration (yes or no) and quality cutoff (25 or 30) as factors, only the ampliconic segment accounted for significant variation in heteroplasmy levels (*P *< 0.001, type III sums of squares). This was consistent with some variation in sample mixing ratios between amplicons. Recalibration and quality cutoff were insignificant (*P *> 0.10) whether or not ampliconic segment was included in the model. Therefore, we achieved a reasonable level of precision in our estimates of heteroplasmy without the need for score recalibration.

### Heteroplasmies in the three families

**Figure 3:**
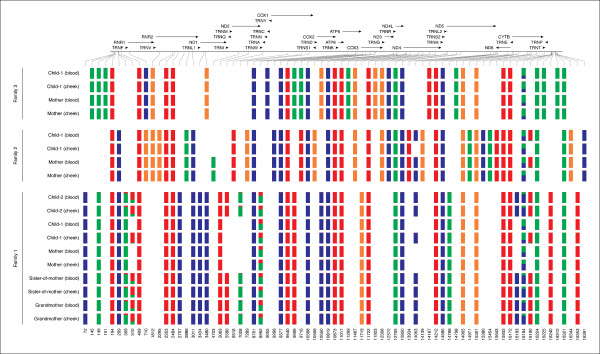
**A representation of all differences found between each sequenced individual and the reference human mtDNA from genome build hg19**. The colored bars (blue = A, green = C, orange = G, red = T) represent the frequency of a given allele in each sample. For example, at position 8,992 one can clearly see a heteroplasmy with two high frequency alleles C and T. Lines on top of the image represent location and orientation of mitochondrial genes. F1 = Family F4, F2 = Family F7, F3 = Family F11.

Using the above criteria, we first identified all sites in our samples that contained differences from the reference with frequency ≥0.02. Note that this initial screening identified not just heteroplasmic sites (which, by definition, must contain two alleles) but also differences between our samples and the reference mtDNA genome (AF347015). A summary representing all such sites is shown in Figure 3. One can see that there is substantial variation among the three families. A *bona fide *heteroplasmic site is evident at position 8,992 in family 4 with two high frequency alleles: C (green) and T (red). To identify heteroplasmies with lower frequencies of the minor allele, we scanned all positions shown in Figure 3 to locate sites containing two allelic variants with frequency ≥0.02. While performing this analysis, we excluded low-complexity regions (66 to 71, 303 to 309, 514 to 523, 12,418 to 12,425, 16,184 to 16,193) for reasons that we explain in the next section. This yielded four sites (including site 8,992 mentioned above) in two of the three families (there were no heteroplasmic sites in family 11) that either showed consistent heteroplasmy in all individuals or exhibited patterns of somatic or germline alterations (Table 1). There was no overlap between the heteroplasmic sites identified in these families and those reported by [16,17] and most recently by the 1000 Genomes Project [23]. The identified sites were divided into three categories: (1) sites without allele frequency shifts; (2) sites with allele frequency shifts and (3) sites with *de novo *mutations (labeled as WS, FS and DN in Table 3, respectively). An extensive search of the MitoMap database and literature revealed that all sites reported here (with the exception of 8,992) have been previously observed as variable, yet only one, 14,053 is non-synonymous.

**Table 1:** Allele frequencies at heteroplasmic sites in Family F4.

			Family F4																								
Tissue	Site	Ref	M5G (grandmother)					M9 (daughter of M5G)					M4 (daughter of M5G)					M4-C1 (child of M4)					M4-C3 (child of M4)				
			A	C	G	T	cvrg	A	C	G	T	cvrg	A	C	G	T	cvrg	A	C	G	T	cvrg	A	C	G	T	cvrg
blood	5063	T	0.000	0.001	0.000	*0.998*	81,207	0.000	0.001	0.000	*0.999*	21,069	0.000	0.016	0.000	*0.984*	12,376	0.000	0.001	0.000	*0.999*	5,228	0.000	0.001	0.000	*0.999*	50,019
	7028	T	0.002	*0.975*	0.001	*0.021*	5,739	0.001	*0.966*	0.001	*0.032*	1,671	0.000	*0.975*	0.000	*0.025*	5,102	no data	0.002	*0.910*	0.000	*0.088*	4,036				
	8992	C	0.000	*0.652*	0.000	*0.347*	52,519	0.000	*0.659*	0.000	*0.341*	15,597	0.000	*0.672*	0.000	*0.327*	14,174	0.000	*0.526*	0.000	*0.474*	4,585	0.000	*0.670*	0.000	*0.330*	35,005
																											
			*A*	*C*	*T*	*G*	cvrg	*A*	*C*	*T*	*G*	cvrg	*A*	*C*	*T*	*G*	cvrg	*A*	*C*	*T*	*G*	cvrg	*A*	*C*	*T*	*G*	cvrg
cheek	5063	T	0.000	0.001	0.000	*0.999*	59,896	0.000	0.001	0.000	*0.999*	20,635	0.000	*0.020*	0.000	*0.980*	2,294	0.000	0.002	0.000	*0.998*	2,073	0.000	0.001	0.000	*0.998*	29,013
	7028	T	0.001	*0.982*	0.001	0.015	3,905	0.001	*0.965*	0.001	*0.033*	1,526	no data					no data					0.001	*0.965*	0.000	*0.034*	2,071
	8992	C	0.000	*0.545*	0.000	*0.454*	38,968	0.000	*0.639*	0.000	*0.360*	14,624	0.000	*0.686*	0.000	*0.314*	1,931	0.001	*0.578*	0.000	*0.421*	1,433	0.000	*0.669*	0.000	*0.330*	19,214

The frequencies were calculated by dividing the number of reads supporting a given allele by the quality adjusted coverage listed in "coverage" column. Quality adjusted coverage = number of reads where the base aligning over a given position has a phred score equal or higher than 30.

The most abundant type of heteroplasmy in our data is the frequency shift (see Figure S4 in Additional file 1 for validation with allele-specific PCR), with site 8,992 in family 4 being the most prominent. Here the major allele frequency fluctuated from a minimum of 0.526 to a maximum of 0.688. Interestingly, in the grandmother (individual M5G; Figure 1) there was a significant (*P <*0.0001, odds ratio test) variation in frequency between blood (C = 0.652 (34,253 reads); T = 0.347 (18,246 reads)) and buccal tissue (C = 0.545 (21,243 reads); T = 0.454 (17,709 reads)). This variation between tissues becomes less profound in one daughter (M9; *P *= 0.0004) and disappears altogether in the other (M4; *P *= 0.96), reappearing in one child of M4 (M4-C1; *P *= 0.0006) but remaining non-significant in the other (M4-C3; *P = *0.98). Only one heteroplasmy (position 5,063; C is the minor allele, G is the major allele) appears to be suggestive of a germline origin. It is observed in blood (the frequency in blood is 0.016, just below the 0.02 error threshold) and buccal tissue (with frequency of 0.0201) of individual M4 (Figure 1). Although other members of family 4 display reads carrying the minor allele, its frequency remains negligible (below 0.001 in all individuals). This includes both children of M4 and suggests that after a *de novo *mutation in M4, the variant allele was lost in her children (we label this loss as a germline allele frequency shift). Two remaining heteroplasmies (site 7,028 in family 4 and site 14,053 in family 7) are both consistent with the frequency-shift scenario, yet insufficient coverage in some individuals and tissues (Tables 1 &2) prevents us from observing transmission events without interruption. At site 7,028 the heteroplasmy shift is of somatic origin (it occurred in blood of M4C3), while at site 8992 it is of germline origin (both analyzed tissues of M4C1 have increased allele frequency). These data suggest that the number of heteroplasmic sites per individual is relatively low and that the frequency of heteroplasmies fluctuates considerably through the transmission events (for a quantitative discussion see Conclusions).

**Table 2:** Allele frequencies at heteroplasmic sites in Family F7.

			Family F7									
			M10 (mother)					M10-C2 (child of M10)				
			A	C	T	G	cvrg	A	C	T	G	cvrg
blood	14053	A	*0.975*	0.010	0.012	0.002	403	no data				
												
cheek	14053	A	*0.970*	0.008	*0.023*	0.000	527	*0.968*	0.003	*0.026*	0.003	380

The frequencies were calculated by dividing the number of reads supporting a given allele by the quality adjusted coverage listed in "coverage" column. Quality adjusted coverage = number of reads where the base aligning over a given position has a phred score equal or higher than 30.

### Erroneous heteroplasmies at low complexity regions

**Figure 4:**
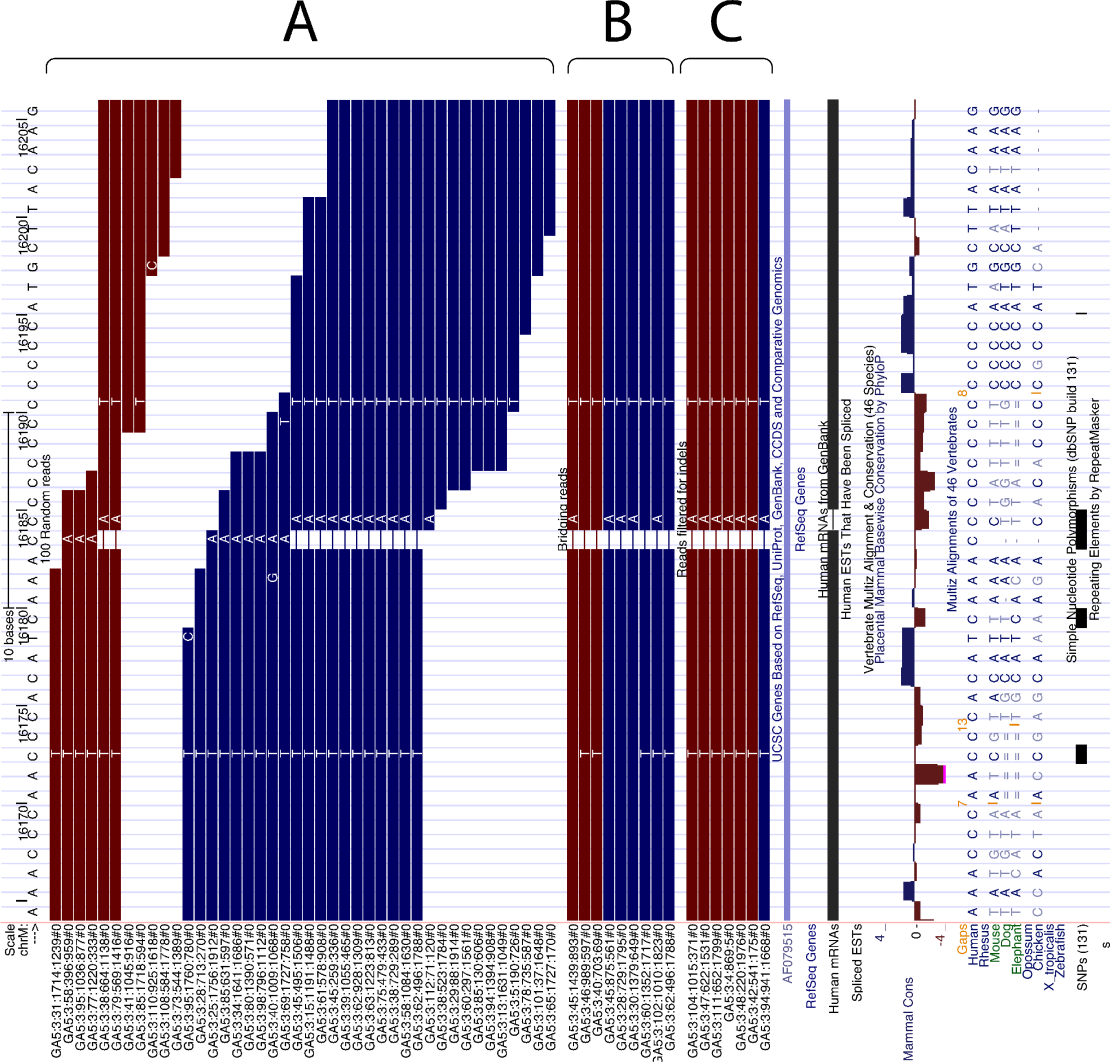
**Reads aligning around the low complexity region 16,184 to 16,190**. Set A: a set of random reads aligning across the region with no quality filtering performed. Set B: bridging reads; these were selected by requiring the low complexity region (positions 16,184 to 16,190) to be in the middle of the read. Set C: high quality reads containing indels; these were required to align across positions 16,184 to 16,190 and contain ten aligning high quality bases (phred value of 30 or higher) on each side of the indel.

Another two sites that immediately stand out in Figure 3 are potential heteroplasmies at positions 309 to 310 and 16,184 to 16,190. They did not make it to the list of heteroplasmies reported here (Table 1) because we excluded low complexity sequences corresponding to these coordinates from the initial analysis. However, the region around site 16,190 has been reported as variable in a number of publications, and most recently He *et al*. [16] highlighted these positions in their re-sequencing of CEPH families. The interesting feature of this region is the fact that it harbors insertion/deletion variation [24-27], and therefore we were interested in examining these sites for possible indel heteroplasmies (note that up to this point we discussed heteroplasmies that involve only point mutations). To do so, we searched for sequencing reads with insertions or deletions relative to the reference sequence using the following stringent approach. For a variant to be called an indel, we required it to be in the middle of a sequencing read and to have ten high quality bases (phred above 30) on each side. Although we did not find sites heteroplasmic for indels using this approach in our samples, we observed that fixed indel polymorphisms might present themselves as erroneous heteroplasmic sites. To illustrate this situation, consider site 16,186, which was initially deemed by us to be heteroplasmic in all individuals examined in the study (Figure 4). A close examination of this site (Figure 4, set A) shows a series of reads with or without a C deletion at position 16,183. Yet one can see that all reads lacking the deletion end nearby (not reaching the end of the 16,163 to 16,169 poly-C stretch), while reads with the deletion extend through the region. To examine this further, we selected a subset of reads that would cover the region shown in Figure 4 completely. As illustrated in set B of Figure 4, all of these reads contain the gap, yet display some disagreement in the A substitution flanking it. Finally, we processed reads further by requiring ten high quality bases (phred ≥30) to extend in both directions from the gap, as shown in set C of Figure 4. As a result, one can see that there is an A insertion and a C deletion at this region that are fixed. Coincidentally, two of the sites confirming maternally derived heteroplasmy in CEPH family 1377 published by Li *et al*. [16] fall within the region we just described. The authors of the manuscript have kindly provided their data and we were able to re-examine the potential heteroplasmy at positions 16,186 and 16,187 (Table 3 in He *et al*. [17]) by remapping the reads to the mitochondrial genome. As shown in Figure S5 in Additional file 1, the frequencies reported by Li *et al*. [16] have likely resulted from misalignment, as very few reads span the poly-C stretch, and both sites reported by the authors (16,186 and 16,187; Table 3 in [16]) likely represent the same C/T transition event that is in fact fixed in all examined individuals. The only difference between the father and the rest of the family is the addition of an A at site 16,183 (which is coincidentally fixed in all individuals of the three families examined here). This example highlights that when identifying indels from short read data, one needs to pay special attention to the positions of identified variants with a read. This is because most 'variation' in set A in Figure 4 is located within the 3' ends of Illumina reads, which are well known to host the majority of inaccurately called bases (likewise with SOLiD reads; see [28] for an excellent overview of the pros and cons of current NGS technologies).

**Table 3:** Context and effect of alleles observed in the six heteroplasmic sites

					Reference			Mutated			
Position	Type	11 bases prior to mutation site	Reference base	Strand	Codon	Amino acid	Codon position	Codon	Amino acid	S/N	Gene
5,063	DN (germline), FS (germline)	CCGTACAACCC	T	+	cct	Pro	3rd	ccc	Pro	S	NADH dehydrogenase subunit 2
7,028	FS (somatic)	TACGTTGTAGC	T	+	ggt	Gly	3rd	ggc	Gly	S	Cytochrome c oxidase subunit I
8,992	FS (germline)	AACCAATAGCC	C	+	ctg	Leu	1st	ttg	Leu	S	ATP synthase 6; ATPase subunit 6
14,053	WS	ACCAAATCTCC	A	+	acc	Thr	1st	ccc	Pro	N	NADH dehydrogenase, subunit 5

DN, de novo mutation; FS, allele frequency shift; WS, without allele frequency shift; N, nonsynonymous **substitutions**; S, synonymous substitutions.

### Replicating our results: a general workflow for the analysis of heteroplasmy

Above we described our methodology for detection of heteroplasmic sites. The same procedure may be useful for other groups studying mitochondrial variation or similar types of mixed samples (for example, viral isolates where frequency of individual variants may vary widely). The second objective of this work was to make our approach easily repeatable so that any reader of this manuscript can reproduce our results or adopt our procedures for use on their own datasets. This is especially relevant as heteroplasmies may be used as potential cancer biomarkers [16,29] and providing the ability to replicate this analysis by any researcher or clinician would therefore be highly beneficial. There are two components to making research reproducible. First, one needs to make data accessible, which is a challenge in itself as some of the datasets generated by NGS technologies are extremely large. Second, one needs to capture all details involved in the analysis of these data, including the tools used and their exact settings. Previously we have developed a software framework - Galaxy [30-32] - that is well suited for disseminating the data and linking them with the analysis tools in a simple to use web-based interface. We used Galaxy to store all the data and to perform all analyses described here.

#### Data

The 32 Illumina datasets representing the three families as well as the pUC18 re-sequencing data are available at Galaxy [18] in addition to being deposited in standard repositories (Sequence Read Archive (SRA), see Materials and methods for accession numbers). From there the datasets can be freely downloaded and readily used to replicate the analyses described in this manuscript.

#### Analyses

**Figure 5:**
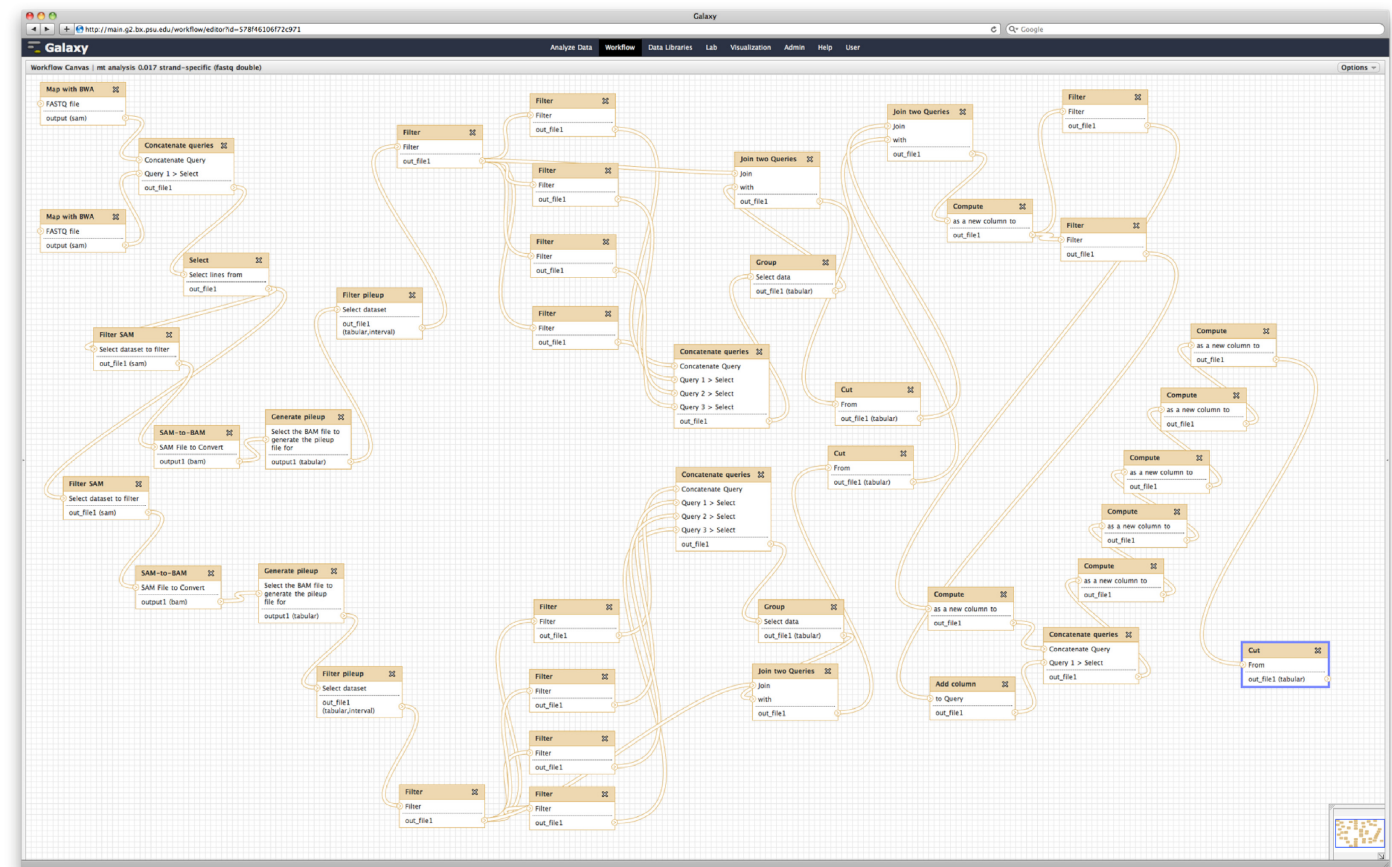
**Workflow for finding heteroplasmic sites from Illumina data**. This workflow can be accessed, used, and edited at [18].

Earlier we described a set of criteria for the detection of heteroplasmic sites. Although these criteria are straightforward, a substantial number of intermediate steps are required to execute them to transform a collection of sequencing reads into a list of heteroplasmies. The Galaxy workflow incorporates all the necessary procedures needed to achieve this (Figure 5). A detailed description of the workflow, links to all analyses we performed to generate Figure 3, Table 1, and Table 2, and a movie explaining minute details of the entire procedure are provided in a dedicated Galaxy page [18] (a Galaxy page is a medium designed to capture all data and metadata associated with a biological analysis [32]). From this page the workflow can be executed as is or modified by anyone, making our analysis completely transparent down to minute details. Briefly, the workflow starts with the sequencing reads, maps them using BWA mapper [20], splits the results into two strand-specific branches (one for the plus strand and one for the minus strand), transforms datasets from read-centric (Sequence Alignment/Map (SAM)) to genome-centric form (pileup) and performs a number of filtering and thresholding steps before merging the branches and generating a list of sites that contain allelic variants with the frequency above 0.01 (at [18], one can click on every step to see the exact set-ting used and a detailed annotation explaining why a particular step was necessary). It is important to note that despite the apparent simplicity of the procedure, a large number of steps is involved (the workflow contains 45 steps) and some of the steps (such as mapping, which is best performed on a multi-CPU machine) require dedicated computational resources. This complex logistics is what creates a formidable wall preventing an 'average' biomedical researcher from performing analyses of NGS data on a regular basis. To the best of our knowledge, this is one of a few re-sequencing studies that publish all data and analyses in a fully reproducible form.

### Repeating the same analysis on the Cloud

Using the workflow provided above, anyone can precisely reproduce the analysis described here, or apply the approach to new datasets. The public Galaxy site [30] (where the workflow is hosted) could be used for this purpose, although this may not always be appropriate for several reasons. First, privacy concerns might prevent the use of an external web resource for processing clinical samples. Second, the public Galaxy site is a heavily used shared resource; if the number or size of datasets to process is considerable, the delays associated with sharing bandwidth and compute resources may not be acceptable or desirable.

An alternative approach is to run a Galaxy instance locally (see [33] for details). Galaxy can easily be installed on a variety of platforms, and workflows can be moved between Galaxy instances. However, this would require acquiring and maintaining local compute resources for Galaxy to use. To perform analysis as quickly as possible would require significant local resources; however, the cost of these resources, particularly if they are not being fully utilized all the time, may be prohibitive.

A very attractive third option is to acquire the compute resources necessary to perform the analysis on demand from a 'cloud computing' provider. This approach is particularly suitable for analyses that benefit from the availability of large amounts of computing power when running, but that are run relatively infrequently. This provides a very cost-effective solution for smaller labs. However, cloud computing resources are typically provided as internet accessible virtual machines, and users must still have informatics expertise to configure and run analysis on them. To address this, we have developed a solution that allows users to quickly deploy and configure a private Galaxy instance on the Amazon AWS cloud using nothing but a web browser (E Afgan *et al*., in press). Additional computational resources can be added to and removed from the private Galaxy instance dynamically, allowing users to perform their analysis as quickly as possible, but only paying for the amount of computing time they use. Combined with the workflow outlined here, this provides a turnkey solution for identifying heteroplasmic sites that is ready to run with nothing but a web browser. In addition, all of the data used here have been deposited into the AWS cloud, allowing readers to exactly reproduce and verify our results. The Galaxy page [18] provides all details for immediate instantiation of an instance capable of repeating all analyses described here (along with the 32 sequencing datasets).

## Conclusions

### Heteroplasmies are relatively infrequent

The first study utilizing NGS technology for detection of heteroplasmies [16] concluded that these events are more frequent than was originally anticipated, with 40 heteroplasmies identified in 10 individuals (using a 1.6% detection threshold). A subsequent study by Li *et al*. [17] utilized a more sophisticated approach and detected 37 heteroplasmies in 131 individuals (using a 10% detection threshold). Li *et al*. used a re-sampling test to demonstrate that they in fact detect significantly fewer heteroplasmies than He *et al*. [16], which may be due to several methodological and/or experimental design issues, such as the source tissues used to isolate mtDNA and the age of studied individuals. Our results are not directly comparable to these two studies because our individuals are related. To make our data compatible with those of Li *et al*., we chose a single individual from each family (M4, M10, and M15 from families 4, 7, and 11, respectively; Figure 1) and counted heteroplasmies above the detection threshold of 10%. This yielded three individuals with a single heteroplasmy in just one of them (at position 8,992 of individual M4; Table 1). This number of heteroplasmic sites (one in three individuals) is not significantly different from the one reported by Li *et al*. (37 among 131 individuals; *P = *0.8375 obtained by simulating 10,000 draws from Poisson distributions with means 37 and 1). The most directly comparable heteroplasmy occurrence in the He *et al*. [16] study is for parents of the two studied families: ten heteroplasmies were observed in four individuals, using the 2% threshold. This is again not significantly different from our observation, with four heteroplasmies in three individuals, at the 2% threshold (*P = *0.4992 obtained by simulating 10,000 draws from Poisson distributions with means 10 and 4). Despite substantial differences in heteroplasmy occurrence, we cannot conclude that this difference is significant, due to the small scale of our and He *et al*.'s [15] studies. To the extent that differences are observed between studies, these may also be attributable to sampling and/or experimental design discrepancies among the three studies resulting in different outcomes, as menrioned above. These considerations led us to be cautious and reluctant to conclude that NGS-based studies will reveal unprecedented numbers of heteroplasmies, even while they are well suited to detection of low frequency heteroplasmies (as described in the introduction). Additionally, the 1000 Genomes Project has identified 67 heteroplasmic sites with frequency above 10% in 163 individuals [22], a number roughly comparable to that of Li *et al*. [17] and this study.

### Heteroplasmy frequency changes through transmission events

Because mitochondria undergo a bottleneck during oogenesis, it is expected that the frequency of alleles at heteroplasmic sites will be different even among related individuals. Site 8,992 in family 4 (Figure 1; Table 1) allows us to test this assumption. This site is heteroplasmic in all five representatives of this family and can be tracked through four transmission events (M5G → M9, M5G → M4, M4 → M4-C1, and M4 → M4-C3; Figure 1). To test whether the allele frequencies are different in each tissue at each transmission event, we performed a re-sampling test using maternal allele frequencies as the background distribution from which we randomly sampled N alleles, where N was equal to the sequencing read coverage in the child in each case. Each re-sampling was performed 10,000 times to construct a distribution from which empirical *P*-values were calculated. Only in one case (M4 → M4-C3) was there no significant difference between frequencies in mother and child (*P = *0.76 and *P *= 0.63 for blood and cheek, respectively; alternative testing using Fisher's exact test for count data gave the same conclusion). These results suggest that the allele frequency at heteroplasmic sites undergoes significant changes during transmission events, and care should be taken when using heteroplasmies as biomarkers in, for instance, forensic or cancer applications. However, these results are based on a single site, two tissues, and a limited number of transmission events. A larger scale study is currently underway in our laboratory, which will help to address these deficiencies.

### A general approach for detection of variants in mixed samples

Detection of heteroplasmies is just one example of a general scenario in which one desires to count variants within a large population of DNA molecules where the frequency of each variant can range from 0 to 1. The approach described here can be used in other cases with one of the most relevant applications being re-sequencing of bacterial or viral populations where distinct isolates are sequenced to identify variants with different phenotypic manifestations [21,34-38]. (Note however, that this is different from analyses of pooled population samples such as those pioneered by Van Tassell *et al*. [39] and perfected by Bansal and colleagues [40,41] in that in these cases the number of pooled individuals is known, allowing expected allele frequencies to be estimated). As bacterial and viral genomes are generally modest in size, an exceptional depth of coverage can be achieved in these cases, significantly reducing the lower bound of detectable allele frequency. Additionally, our methodology can be further improved by using information about positions of variant bases within sequencing reads, as was proposed by Bansal *et al*. [41], and adding tools for haplotype reconstruction previously implemented by our group [21] or most recently proposed by Zagordi *et al*. [42].

### A turnkey solution for re-sequencing of mixed samples

As was noted in the Results and discussion, reproducibility is the Achilles' heel of modern life sciences. Even the two manuscripts most frequently mentioned here - He *et al*. [16] and Li *et al*. [17] - are not entirely reproducible as sequencing data are only available on request and the exact settings of tools used and some of the scripts utilized in the data processing are not available as supplementary material. We emphasize that in highlighting these deficiencies we are not being critical of these authors, as making data, tools, and research metadata universally accessible is an engineering challenge in itself. To establish a precedent of data- and computationally intensive re-sequencing studies being completely reproducible, we leveraged the Galaxy system [32] to make all data and analysis steps accessible and transparent. Importantly, anyone possessing similar datasets can use our workflow to analyze their own data through the Galaxy public service [18], their own installation [33], or using Amazon Cloud [43] for a complete 'hardware-free' solution. This makes our work completely transparent and re-usable as anyone has complete access to all analytical details and can modify our protocol and adopt it to his/her needs. It is our hope that Galaxy, together with developing analysis portals such as MyExperiment [44] and Genomespace [45], will be able to significantly increase the number of fully reproducible studies in the biomedical sciences.

## Materials and methods

### Samples

Several families were recruited in this study; however, for three families (4, 7, and 11; Figure 1) we were able to amplify mtDNA (see below) in sufficient quantities first and thus samples from these three families were used for subsequent sequencing and analysis. Blood and cheek swab were obtained with informed written consent from each individual. This study was approved by the Human Subjects Protection Office of the Penn State College of Medicine.

### Sample collection and DNA extraction

Blood was collected from a finger using a BD Microtainer contact-activated lancet (catalogue number 366593 or 366594; BD, Franklin Lakes, NJ, USA) and was preserved in BD Microtainer Tubes with K2E (catalogue number 365974) until DNA extraction. DNA was isolated using Qiagen DNeasy Blood and Tissue Kit (Qiagen Sciences, Germantown, MD, USA). Finally, DNA was placed in 200 mL Tris-EDTA (TE) buffer (10 mM Tris-HCl, 1 mM EDTA, pH 8.0).

DNA extraction from buccal cells was carried out according to the method detailed in Freeman *et al*. [46]. Buccal cells were collected by scraping the inside of the mouth with cotton swabs on plastic sticks. These swabs were placed in Slagboom buffer (0.1 M NaCl, 10 mM Tris-HCl pH8, 10 mM EDTA, 0.5% SDS) with Proteinase K (0.2 mg/ml). Proteins were removed by organic de-proteinization reagent (ORPR), and DNA was precipitated with isopropyl alcohol. The DNA was re-suspended in 250 ml of TE buffer.

### PCR amplification

Whole mitochondrial DNA was amplified with two sets of primers: L2815 and H11571; L10796 and H3370. These primers were originally described in Tanaka *et al*. [47]. The PCR amplification was performed in 20 μl with 10 ng genomic DNA, 0.2 mM dNTPs (PCR grade; Roche Applied Science, Indianapolis, IN, USA), 0.84 units Expand High Fidelity PCR Enzyme mix (Roche Applied Science), 1 × buffer including 1.5 mM Mg^2+^, and 0.4 μM forward and reverse primers (Integrated DNA Technologies, Inc., Skokie, Illinois, USA). Thermal cycling conditions consisted of two different cycles. The first cycle was 94°C for 15 s, 60°C for 30 s, and 68°C for 8 minutes for 10 repeats. The second cycle was 94°C for 15 s, 60°C for 30 s, and 72°C for 8 minutes for 20 repeats. The extension time was elongated by 5 seconds for each successive cycle. The PCR product was cleaned-up by gel purification with NucleoSpin Extract II kit (Macherey-Nagel GmbH and Co. KG, Düren, Germany). For each sample, two PCR products obtained by two independent reactions were prepared for sequencing.

### Preparation and sequencing of clonal DNA

AG1 cells (50 μl) were heat-shock transformed (42°C, 45 s) with 1 pg pUC18 DNA (catalogue number 200232, Agilent Technologies, Santa Clara, CA, USA). AG1 cells were chosen because they are endonuclease (endA) and recombination (recA) deficient, but also because they lack an episome, which might contaminate plasmid preparations. A reduced DNA input was used (kit suggests 100 pg pUC18 into 100 μl) to reduce sample variability by minimizing the risk of double-transformants. A single colony was picked and grown in 300 ml LB to an OD600 of approximately 0.6 (approximately 13.5 hours) and DNA extracted from half this volume using the EndoFree PerfectPrep Maxi kit (catalogue number 7855475, 5 Prime, Gaithersburg, MD, USA; supplemental RNase A was added to the lysis buffer to increase the concentration from 0.5 mg/ml to 1 mg/ml). Ampicillin was maintained at 100 μg/ml in plates and liquid cultures. DNA purity and concentration were examined by nanodrop spectroscopy, gel electrophoresis and PicoGreen quantification (the latter two in approximate agreement). DNA sequencing was performed at Sequensys (La Jolla, CA, USA; a division of Prognosys Biosciences, Inc.) by the same method described below.

### Assessment of variation in clonal DNA

The same pUC18 DNA that had been subject to Illumina sequencing (procedure described above) was transformed again (1 pg in 50 μl AG1 cells) and 192 sub-clones were sequenced using the Sanger method for which the primer PSU18-F (5'-GGCGCTTTCTCATAGCTCAC-3'; covering bases 1,049 to 1,068) was used. Sanger sequences were visualized using the Staden package and 691 bases of quality-trimmed sequence were identified as invariant in 191 clones (one clone failing to yield high-quality sequence along the full length). After subcloning and sequencing a further 192 clones, 607 bases of quality-trimmed sequence were identified in 186 clones (six clones failing to yield high-quality sequence), providing strong evidence for invariance across the region.

### Preparation of mixed samples

To further assess the accuracy and precision of our polymorphism detection, we prepared a sample by mixing DNA from two individuals described in the main dataset (M4 and M10C1) in an approximately 49:1 ratio. At sites with fixed differences between these individuals, this procedure was expected to yield a 2% apparent minor allele frequency with the identity of the minor allele corresponding to the M10C1's sequence. For the mixing procedure we handled each amplicon (A and B) separately, attempting to add 490 ng of M4 DNA to 10 ng of M10C1 DNA. First, DNA concentrations for all samples were estimated by nanodrop spectroscopy, and second, M10C1 DNA was diluted and the dilution's DNA concentration was estimated. This procedure allowed us to add DNA from both individuals in a 49:1 ratio using a single pipette (a Gilsen P10), thereby reducing pipetting error (which we estimate to be approximately 2 to 4%).

### Sequencing and analysis

#### Sequencing

DNA sequencing was performed at Sequensys on an Illumina GA IIx instrument (software version 1.8) with multiplexing (12 samples per lane). All datasets generated within this study are accessible for immediate download and analysis as described at [18] (the datasets and workflows are also available directly from the Amazon Cloud at [48]; Illumina reads may also be download from SRA at NCBI (project ID 67461, submission DRA000390, study DRP000396, samples DRS000673 to DRS000684, DRX000679 to DRX000701, DRR001058 to DRR001100]).

#### Identification of heteroplasmic sites

A complete workflow for identification of heteroplasmic sites is shown in Figure 5 and can be accessed, viewed, and edited at [18] (in addition, the exact settings of each tool can be viewed at that site). It uses BWA mapper (version 0.5.6) [20] for initial mapping of reads, SAMtools [49] for processing of generated SAM datasets and a collection of Galaxy tools for transformation and filtering of data. A screencast (short narrated movie) at [18] explains how the workflow can be used for the analysis of multiple datasets.

#### Allele-specific PCR

Allele-specific PCR amplification was performed with 5 μl of 100 diluted ampliconic DNA (from amplicon A; for site 7,028) or 2 μl genomic DNA (for site 8,992). Also added were 0.2 mM dNTPs, 0.5 μM forward and reverse primers (Integrated DNA Technologies, Inc.), 1 × buffer including 1.5 mM Mg^2+^, and 2 units of Choice Taq (Denville Scientific Inc., Metuchen, New Jersey, USA), all diluted to 50 μl with PCR-grade water (Teknova Inc., Hollister, CA, USA). Forward primers were designed to amplify each allele specifically with the 3' end nucleotide adjusted accordingly and the nucleotide in the -1 position also changed to further destabilize the duplex (after the strategy described in Figure 3 of [50]; although note that 7,028 primers are designed for the reverse strand). For each locus a common reverse primer was included for amplification. Primer pairs were checked by reverse ePCR [51] against human reference genome assembly 37.1 to reduce the risk of amplification from numts, with reported pairs showing no hits. For site 7,028, thermal cycling conditions consisted of 94°C for 45 s, 60°C for 30 s, and 72°C for 3.5 minutes for 30 cycles. For site 8,992, the thermal profile was 94°C for 45 s, 55°C for 30 s, and 72°C for 3 minutes also for 30 cycles. For both sites this was preceded by 94°C for 3 minutes and followed by a terminal exten-sion step at 72°C for 10 minutes.

### Simulations

A FASTA file is read into a string object and empty reads are created at random intervals across its length (a python script performing this analysis is available as Additional file 3). These reads consist of lists of indices corresponding to positions in the sequence string allowing the program to account for circularity by creating some discontinuous lists (spanning the origin). Next, sublists within a list object (colloquially known as the quasispecies 2D list) are populated using read indices to recover bases from the sequence string. At a randomly chosen index, corresponding to the heteroplasmic site, this process is modified by passing bases through a dictionary that substitutes A/G and T/C bases, but this is done with a probability equal to the user-specified minor allele frequency. At all positions the recovered base is also passed through an error dictionary that substitutes A/C and T/G bases with a probability equal to the user-specified error rate (0.001 in this study). Finally, the program examines the quasispecies list to extract information on false positives and false negatives using the user-specified frequency cutoff. At each index in the quasispecies list (corresponding to a genome position) the sum of each base type within the sublist is assigned to a dictionary together with the length of the sublist (read coverage). Next, the key and value corresponding to the reference base is deleted and the maximum read count is extracted from the remaining three entries and divided by the coverage to yield the maximum variant frequency. If this exceeds the user-specified cutoff, a false positive variable is incremented. At the heteroplasmic base the key corresponding to a minor allele (for example, a G if the reference is an A) is first examined and a false negative variable is incremented if this (divided by coverage) is less than the threshold. Finally, these variables and the genome size (the length of the sequence string) are printed to a tab-delimited text file.

## Abbreviations

EST: expressed sequence tag; mtDNA: mitochondrial DNA; NGS: next-generation sequencing; PCR: polymerase chain reaction; SNP: single-nucleotide polymorphism; SRA: Sequence Read Archive.

## Competing interests

The authors declare that they have no competing interests.

## Authors' contributions

KDM, JT and AN conceived and supervised the project. HG and BD and performed the experiments and some of the statistical analyses. EA and JT implemented major components for Cloud deployment. AN and KDM wrote the paper. All authors contributed to testing, data analysis, and the writing of the manuscript. All authors reviewed and approved this manuscript.

## Supplementary Material

Additional file 1 **Supplemental Figures S1, S2, S3, S4, and S5**. Click here for file

Additional file 2 **Supplemental Table S1**. Click here for file

Additional file 3 **FN-FP-simulation-script.py**. A script for performing simulation performed in Results and discussion. Click here for file
