# Supplementary material for: Dynamics of mitochondrial heteroplasmy in three families investigated via a repeatable re-sequencing study
Source: Genome Biol. 2011 Jun 23;12(6):R59. doi: 10.1186/gb-2011-12-6-r59 (PMC3218847; doi:10.1186/gb-2011-12-6-r59)
Supplement: Additional file 1 — Supplemental Figures S1, S2, S3, S4, and S5. [file gb-2011-12-6-r59-S1.PDF]

Figure S1

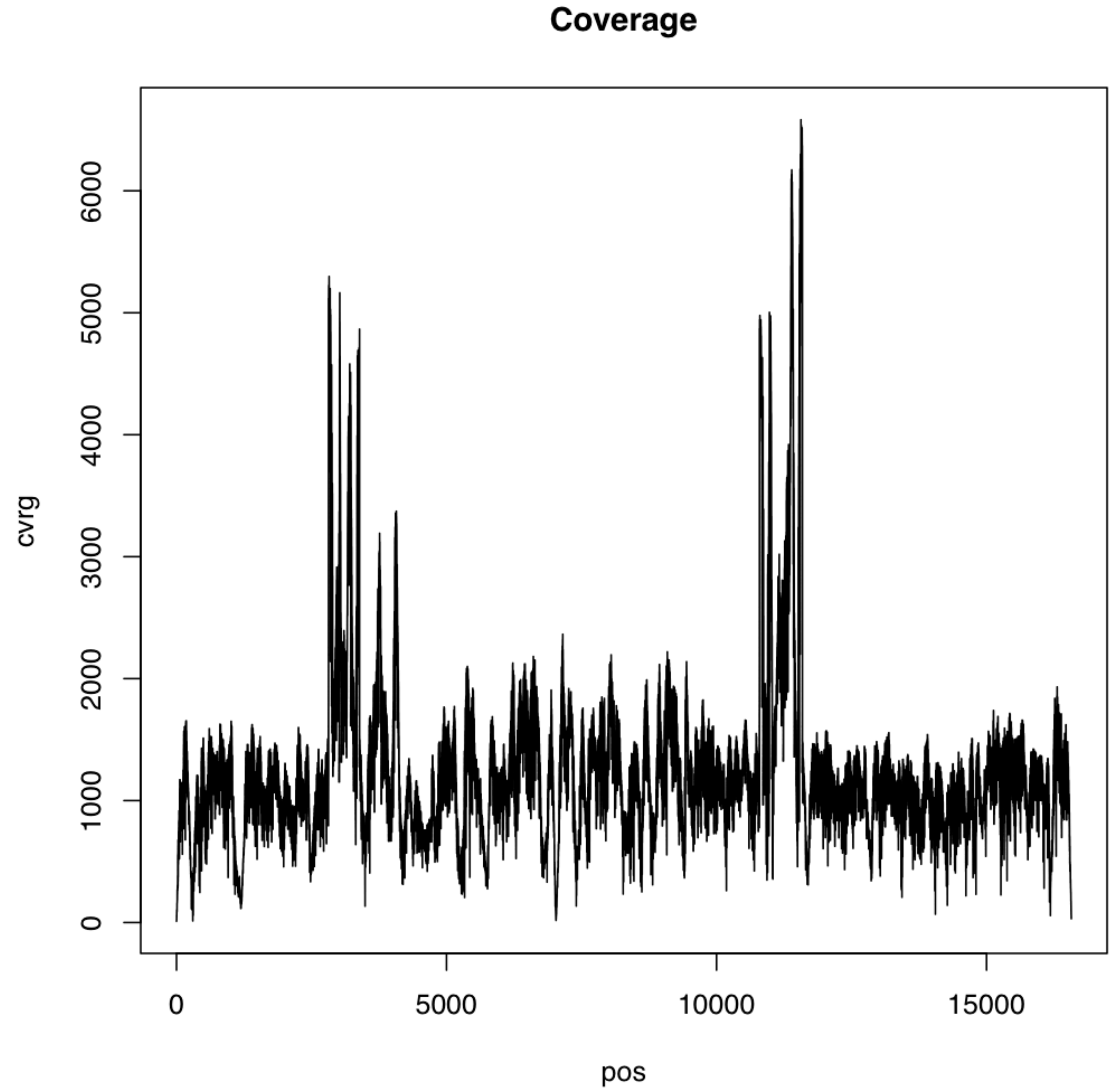

Coverage
cvrg
0
1000
2000
3000
4000
5000
6000
0
5000
10000
15000
pos

Figure S2

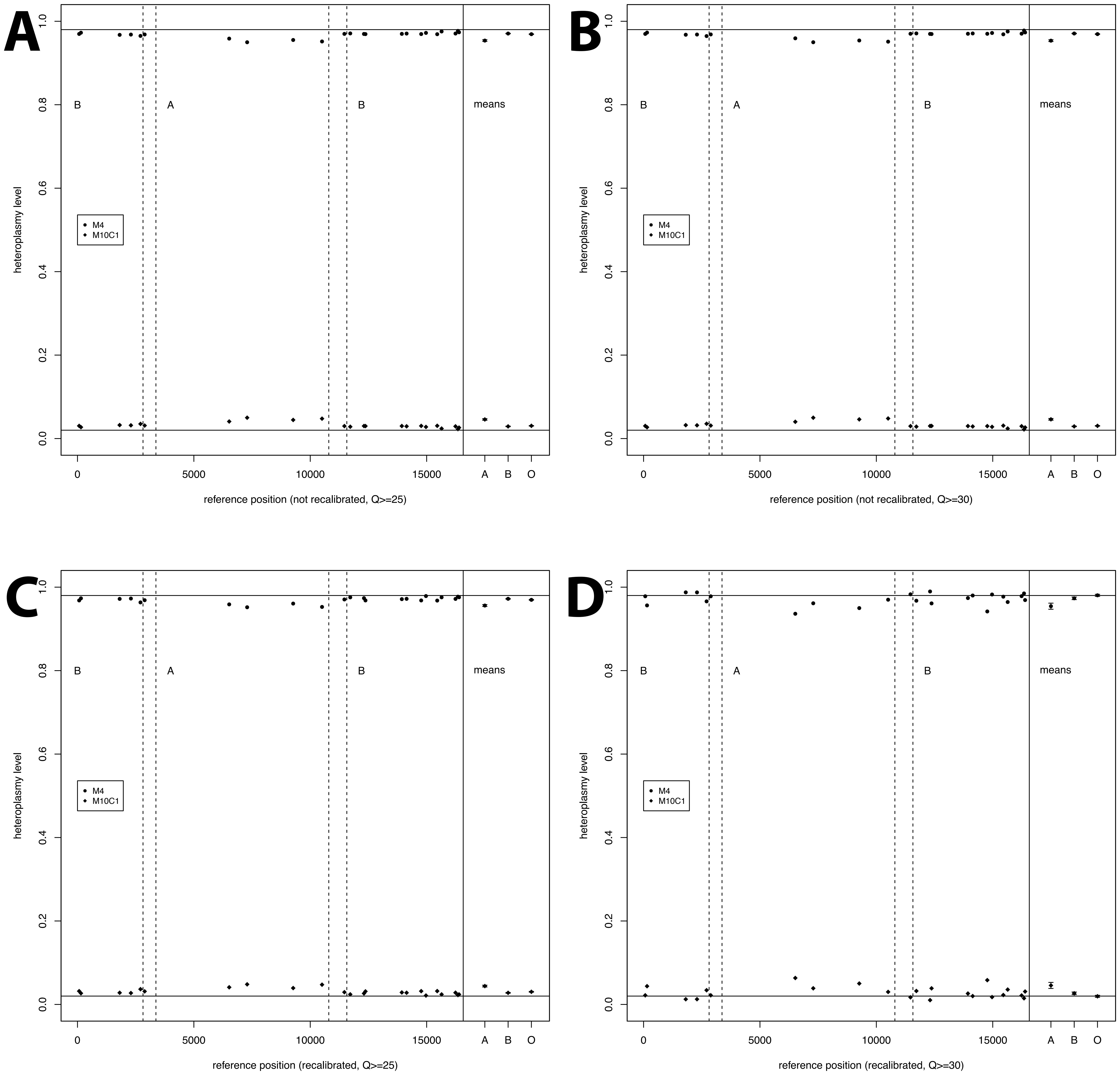
A
B
C
D
heteroplasmy level
B
A
B
means
M4
M10C1
reference position (not recalibrated, Q>=25)
reference position (not recalibrated, Q>=30)
reference position (recalibrated, Q>=25)
reference position (recalibrated, Q>=30)

Figure S3

Mixed sample (49[M4]:1[M10])

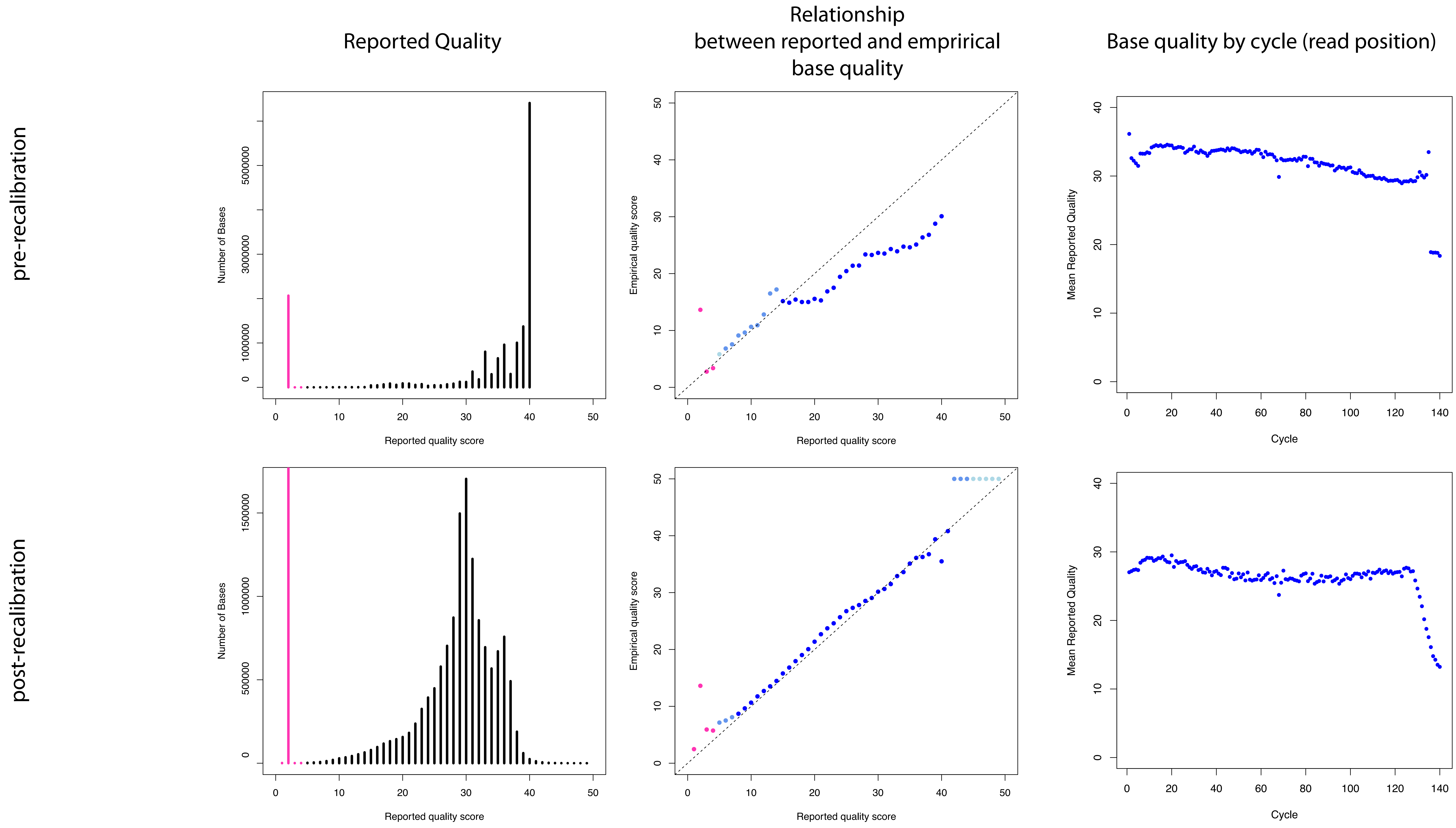

Figure S4

site 7028

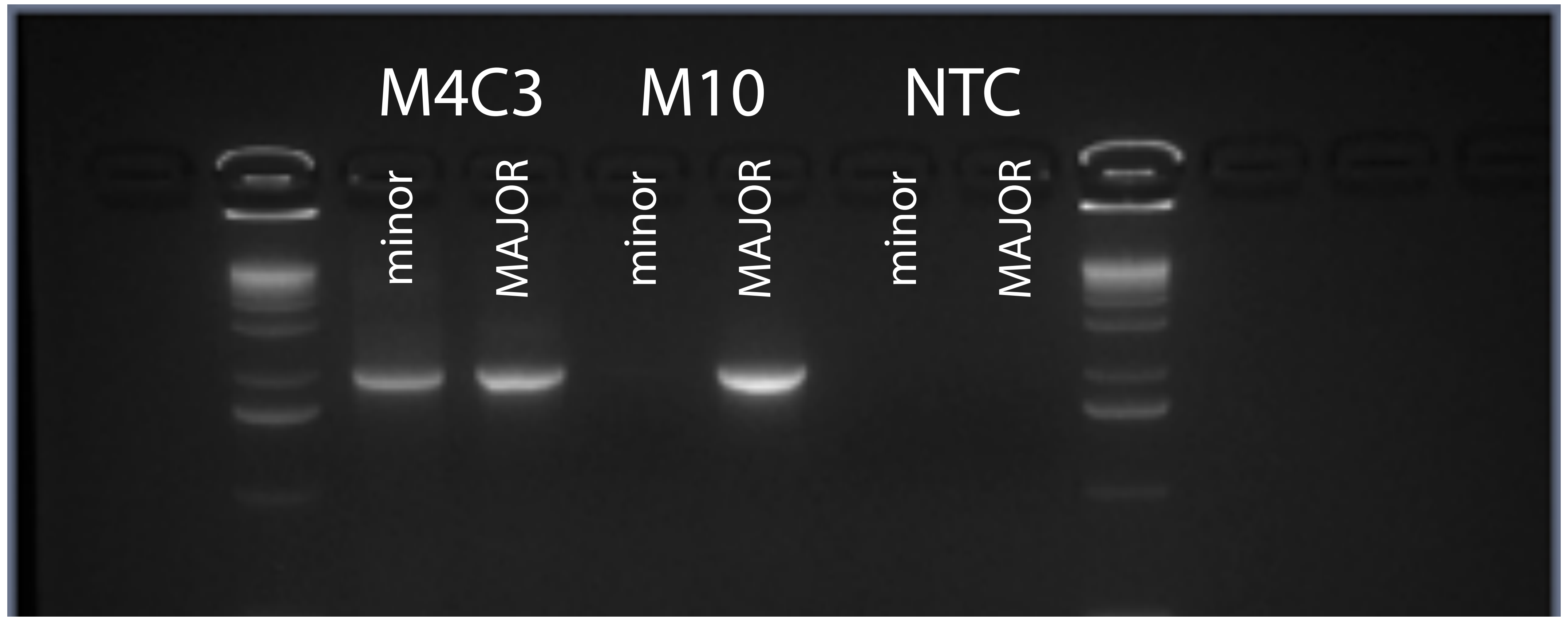

site 8992

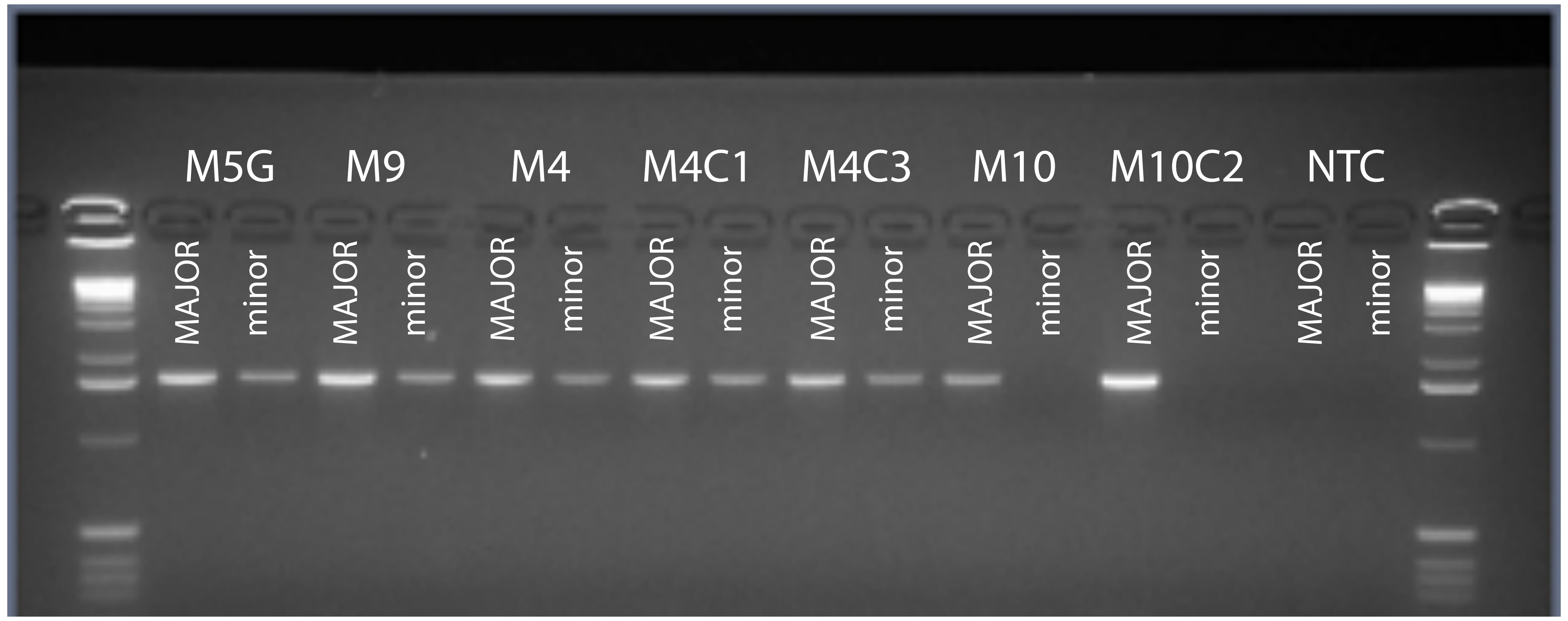

Figure S5

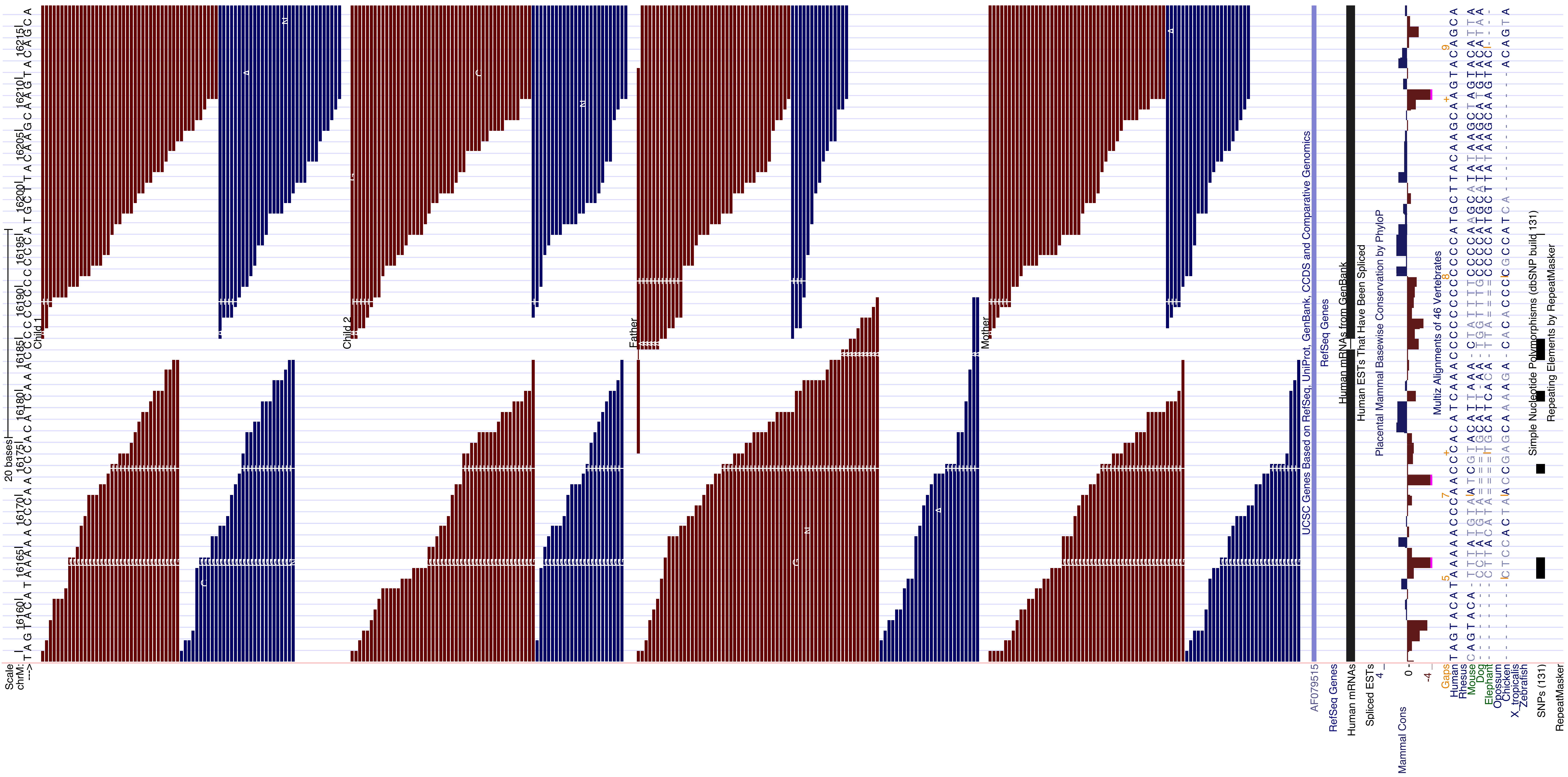

Scale
chrM:
20 bases
Child 1
Child 2
Father
Mother
UCSC Genes Based on RefSeq, UniProt, GenBank, CCDS and Comparative Genomics
AF079515
RefSeq Genes
Human mRNAs from GenBank
Human mRNAs
Human ESTs That Have Been Spliced
Spliced ESTs
Placental Mammal Basewise Conservation by PhyloP
Mammal Cons
Multiz Alignments of 46 Vertebrates
Gaps
Human
Rhesus
Mouse
Dog
Elephant
Opossum
Chicken
X_tropicalis
Zebrafish
Simple Nucleotide Polymorphisms (dbSNP build 131)
SNPs (131)
Repeating Elements by RepeatMasker
RepeatMasker
