# Supplementary material for: Dynamics of mitochondrial heteroplasmy in three families investigated via a repeatable re-sequencing study
Source: Genome Biol. 2011 Jun 23;12(6):R59. doi: 10.1186/gb-2011-12-6-r59 (PMC3218847; doi:10.1186/gb-2011-12-6-r59)
Supplement: Additional file 2 — Supplemental Table S1. [file gb-2011-12-6-r59-S2.PDF]

Table S1. Comparison of base counts and frequencies at two PCR replicated for blood of M4.

Position	Reference Base	Counts									
		PCR1					PCR2				
		A	C	G	T	coverage	A	C	G	T	coverage
5063	T	2	104	1	6687	6794	3	89	2	5488	5582
7028	T	0	2858	0	72	2930	0	2117	0	55	2172
8992	C	2	5455	0	2646	8103	1	4241	0	2097	6339
		Frequencies									
5063	T	0.0003	0.0153	0.0001	0.9843		0.0005	0.0159	0.0004	0.9832	
7028	T	0.0000	0.9754	0.0000	0.0246		0.0000	0.9747	0.0000	0.0253	
8992	C	0.0002	0.6732	0.0000	0.3265		0.0002	0.6690	0.0000	0.3308	
